# Priming strategies to enhance sprint performance in highly trained athletes across sports: a systematic review and meta-analysis

**DOI:** 10.5114/biolsport.2026.161110

**Published:** 2026-05-20

**Authors:** Krzysztof Kotuła, Lucas A. Pereira, Adam Zajac, Artur Golas, Adam Maszczyk, Irineu Loturco

**Affiliations:** 1 Institute of Sport Sciences, Jerzy Kukuczka Academy of Physical Education, Katowice, Poland; 2 NAR – Nucleus of High Performance in Sport, São Paulo, Brazil; 3 Department of Human Movement Sciences, Federal University of São Paulo, São Paulo, Brazil; 4 Football Science Institute, Granada, Spain

**Keywords:** Speed ability, Elite athletes, Athletic performance, Team sports, Sprinting

## Abstract

Priming strategies have been proposed as a practical approach to acutely enhance neuromuscular readiness and improve speed- and power-related performance in athletes. This systematic review and metaanalysis aimed to evaluate the effectiveness of different priming protocols on sprint performance in highly trained athletes across sports. A comprehensive literature search was conducted across PubMed, Scopus, Web of Science, SPORTDiscus, Medline, Science Citation Index Expanded, and Embase databases up to February 26, 2026. Randomized studies investigating the sub-acute effects (≥ 5–48 h) of priming interventions on linear sprint performance in competitive athletes were included. Fifteen studies comprising 262 athletes were analyzed. A random-effects model was used to calculate standardized mean differences (SMD). Overall, priming interventions significantly improved sprint performance (SMD = 0.40; 95% confidence interval: 0.22–0.59; p < 0.001) with low heterogeneity (I^2^ = 5%). Subgroup analysis revealed significant improvements when priming sessions were performed ≤ 8 hours before performance testing, whereas no significant effects were observed for longer intervals (≥ 20 h). No significant differences were detected between subgroups. These findings indicate that low-volume, high-intensity priming strategies (i.e., light-to-heavy load traditional and ballistic resistance exercises) may represent an effective approach to acutely enhance sprint performance in highly trained athletes across sports.

## INTRODUCTION

The development of sprint speed is a critical determinant of performance across a wide range of sport disciplines [1–4]. In both individual and team sports, the ability to accelerate rapidly and achieve high running velocities frequently differentiates competitive outcomes and overall performance levels [1–4]. Despite its relevance, achieving further meaningful improvements in sprint performance remains challenging, particularly in highly trained athletes, in whom neuromuscular adaptations — especially sprint-specific adaptations — tend to plateau and inter-individual performance differences are often minimal or even nonexistent [3, 5, 6]. In this context, even small enhancements in sprint speed may have substantial competitive implications [2–4, 6].

To address these constraints, pre-conditioning strategies have been implemented as potential methods to acutely or sub-acutely enhance speed and power performance [7–10]. In this regard, two distinct approaches have been consistently examined and described in the literature: post-activation performance enhancement (PAPE) and priming interventions. Although these terms are frequently used interchangeably, they represent physiologically and temporally distinct phenomena. In general, PAPE refers to an acute enhancement in performance occurring within approximately 4–15 minutes following a conditioning activity (CA), typically involving high-intensity resistance exercise (e.g., squats at 50–80% one-repetition maximum [1RM]) or ballistic exercises (e.g., jump squats at 30–40% 1RM) [7, 10]. Such enhancements are observed in a variety of neuromuscular tasks, including vertical jumping and sprinting, and are thought to be mediated by mechanisms including increased motor unit recruitment, enhanced phosphorylation of myosin regulatory light chains, and alterations in muscle-tendon stiffness [7, 10]. Nevertheless, these effects are transient and may dissipate within 20–30 minutes post-CA [7, 10]. For this reason, PAPE techniques are commonly used during warm-up routines to maximize speed and power capacities, mainly in sports where explosive actions are of fundamental importance (e.g., sprint and jump events) [7, 11]. Notably, recent meta-analytic evidence suggests that PAPE-related strategies do not substantially enhance sprint performance in elite sprinters [11], indicating that the magnitude of acute potentiation may be attenuated in athletes with highly developed neuromuscular functions and higher relative strength and power levels.

In contrast to PAPE, priming interventions refer to performance enhancements observed after longer recovery intervals, typically ranging from 5 to 48 hours following the CA [8, 10]. Rather than inducing immediate potentiation and performance improvements, priming exercises appear to promote a temporary optimization of neuromuscular readiness that persists beyond the acute recovery window, which may vary according to exercise type and athlete training background [8, 9]. Due to the regular competition schedule, with most competitions occurring during the afternoon or night periods, these protocols are frequently implemented as “morning priming sessions” (5–8 hours pre-competition) or as “day-before priming sessions” (24–48 hours pre-competition), particularly in elite sport environments, where logistical and structural constraints limit access to facilities immediately prior to competition [8–10]. Although the precise mechanisms underpinning priming effects remain incompletely understood, proposed explanations include transient increases in neural drive, modulation of central nervous system excitability, enhanced muscle contractile function, and favorable shifts in the fatigue–potentiation balance [8–10].

Importantly, priming protocols are typically characterized by high intensity (in terms of relative load or movement velocity) and low volume [8, 9], aiming to stimulate neuromuscular activation without generating significant residual fatigue that could impair subsequent speed- or power-related performance. A variety of priming protocols have been investigated, including those involving ballistic and traditional strength-power exercises, plyometrics, and unresisted, resisted, and assisted sprint drills [12–15]. Performance improvements have been reported in speed and power tasks within time intervals ranging from 5 to 48 hours post-intervention [8, 9]. Nonetheless, the available evidence remains inconsistent, with studies differing substantially in athlete population, priming protocol (i.e., exercise type × sets × repetitions), recovery intervals, and performance variables or metrics assessed (e.g., acceleration vs. maximal speed, power output, jump height) [8, 9].

Despite the growing applied interest in priming strategies [16, 17], no systematic review and meta-analysis has comprehensively tested and quantified their effects on sprint performance in competitive athletes, whether from individual or team sports (e.g., track and field, soccer, rugby, etc.). This represents a critical gap in the literature. Given that highly trained athletes exhibit reduced adaptive reserve for sprinting speed (i.e., reduced windows of speed development) and minimal performance differences between individuals, even small increases in sprint speed may have meaningful competitive implications [3, 5, 6]. Therefore, a rigorous synthesis of the available evidence is warranted to determine the magnitude and consistency of priming effects on sprint performance under these conditions. The main purpose of this systematic review was to evaluate the effectiveness of different priming protocols on sprint performance in highly trained athletes across sports. Specifically, we examined studies investigating the sub-acute effects (5–48 hours) of priming interventions on sprint speed and conducted a meta-analysis to quantify the magnitude of these effects.

## MATERIALS AND METHODS

### Study Design

The purpose of this systematic review and meta-analysis was to assess the effectiveness of different priming protocols on sprint performance in highly trained athletes. All procedures performed in this study were in accordance with the “Preferred Reporting Items for Systematic Reviews and Meta-Analyses” (PRISMA) guidelines [18]. The criteria for the literature search and data sources, study eligibility and selection, and data extraction and analysis, as well as the assessment of risk of bias, are presented and described in the following sections.

### Search Strategy

A comprehensive literature search was conducted across the following electronic databases: PubMed, Scopus, Web of Science, SPORTDiscus, Medline, Science Citation Index Expanded (SCI-Expanded), and Embase via Ovid. This search included studies published until February 26, 2026. The search strategy employed a structured query designed to capture all studies relevant to the research question. Medical Subject Headings (MeSH) and free-text keywords were combined using Boolean operators (AND, OR), following the PICOs framework (i.e., participants, intervention, comparator, and outcomes) as follows: (“elite athlete” OR “highly trained” OR “national level” OR “international level” OR “professional athlete” OR “competitive athlete”) AND (“priming” OR “post-activation potentiation” OR “PAP” OR “PAPE” OR “post-activation performance enhancement” OR “conditioning activity” OR “preconditioning” OR “pre-activation” OR “warm-up potentiation” OR “delayed potentiation” OR “morning priming” OR “day-before” OR “pre-competition”) AND (“sprint performance” OR “sprint time” OR “sprint speed” OR “sprint velocity” OR “acceleration” OR “maximal velocity” OR “running performance” OR “sprinting” OR “linear speed”).

### Eligibility Criteria

Randomized peer-reviewed studies published in English only were considered for inclusion, with no age or sex restrictions. Studies were included based on the following criteria: 1) subjects underwent a priming intervention — with a minimal time between the exercise and the performance test of at least 2 hours; 2) the sample was exclusively composed of highly trained athletes with no distinction regarding competitive level; 3) linear sprint performance (i.e., sprinting speed or sprinting time) was defined as the outcome variable. Studies were excluded if: 1) no comparison group or baseline measurement was included; 2) chronic (i.e., long-term) effects were assessed; 3) the full text was not available; 4) ergogenic aids (e.g., ischemic preconditioning, caffeine, sodium bicarbonate, and transcranial direct current stimulation) or psychological strategies (e.g., motor imagery, mental rehearsal, self-talk, and arousal) were utilized.

### Study selection

The search was conducted by three researchers (LAP, AM, and AG). After removing duplicates, titles and abstracts were screened, and studies that did not meet the eligibility criteria were excluded. Subsequently, the full texts of the remaining articles were assessed. Then, independently and in a blinded manner, three reviewers (LAP, AM, and AG) selected the studies for inclusion, following the eligibility criteria. In cases where no agreement was reached, a fourth researcher (IL) was consulted.

### Data Extraction

Mean, standard deviation, and sample size data were extracted from the included manuscripts by three reviewers (LAP, AM, and AG). When needed, the authors were contacted to obtain missing data. If there was no response from the authors, the study was excluded from the analysis. Any disagreements during the process of data extraction and analysis were resolved by consensus among all authors. To standardize data extraction, pre- and post-priming results for the longest sprint distance of each study were recorded and used in the analysis. In addition, general study characteristics, including design, participant demographics (i.e., age, sex, training background, competition level), randomization, and blinding procedures, were also recorded.

### Risk of Bias Assessment

Methodological quality was independently assessed by two reviewers using a modified Cochrane Risk of Bias tool (RoB 2.0) tailored for acute interventions, as recommended by the Cochrane Collaboration guidelines [19]. Domains evaluated included random sequence generation, allocation concealment, assessor blinding, completeness of outcome data, selective reporting, and other biases such as inadequate washout periods in crossover designs or lack of familiarization sessions. Each domain was rated as low, unclear, or high risk of bias. Overall study quality was categorized as low risk (low risk in all domains), moderate risk (unclear risk in one or more domains), or high risk (high risk in one or more domains).

### Statistical Analysis

The analysis was conducted using Microsoft Excel. The primary analysis was performed using a random-effects model based on the DerSimonian and Laird method [20]. The magnitude of the effects was calculated as standardized mean differences (SMD – Hedges *’ g*). When outcomes were reported as time or velocity, the direction of the effect was standardized so that positive values consistently reflected performance improvement. The thresholds used to qualitatively interpret SMD were: < 0.2 (trivial), ≥ 0.2 (small), ≥ 0.5 (moderate), and ≥ 0.8 (large) [21]. For studies including multiple priming protocols within the same sample, effect sizes were combined to avoid a unit-of-analysis error. Combined mean differences were calculated as the arithmetic mean of protocol-specific effects. Variances were pooled while accounting for dependencies between protocols using an assumed correlation coefficient (*r* = 0.5). Sensitivity analyses were conducted by varying the *r* values between 0.3 and 0.7 to examine the robustness of the results. Heterogeneity between studies was assessed using Cochran’s *Q* and quantified using I^2^ statistics [22]. I^2^ values were interpreted as low (< 25%), moderate (25–50%), or high (> 50%) heterogeneity. Between-study variance (τ^2^) was estimated using the same method [20]. In addition to pooled effects, 95% prediction intervals were calculated under the randomeffects model to estimate the expected range of effects in future studies, accounting for between-study heterogeneity. Subgroup analyses were performed according to the time interval between priming exercises and performance testing (≤ 8 hours and ≥ 20 hours). Differences between subgroups were assessed by comparing pooled SMD using a Z test based on the difference between subgroup estimates and their standard errors. Publication bias was evaluated through visual inspection of funnel plot asymmetry and statistically tested using Egger’s regression test; a *p*-value < 0.10 was considered indicative of small-study effects. The statistical significance level for overall effects was set at *p* < 0.05.

## RESULTS

A total of 1308 records were initially identified through database searches, and 18 additional studies were found from other sources. After screening titles and abstracts, 768 of the 818 studies remaining following duplicate removal were excluded. As a result, 50 studies were assessed for eligibility, and based on the inclusion and exclusion criteria, 15 studies were finally included in the meta-analysis (Figure 1) [12–15, 23–33]. The risk of bias analysis among the included studies is presented in Figure 2. Of the 15 studies analyzed, 11 demonstrated low risk and 4 showed high risk of bias.

**FIG. 1:**
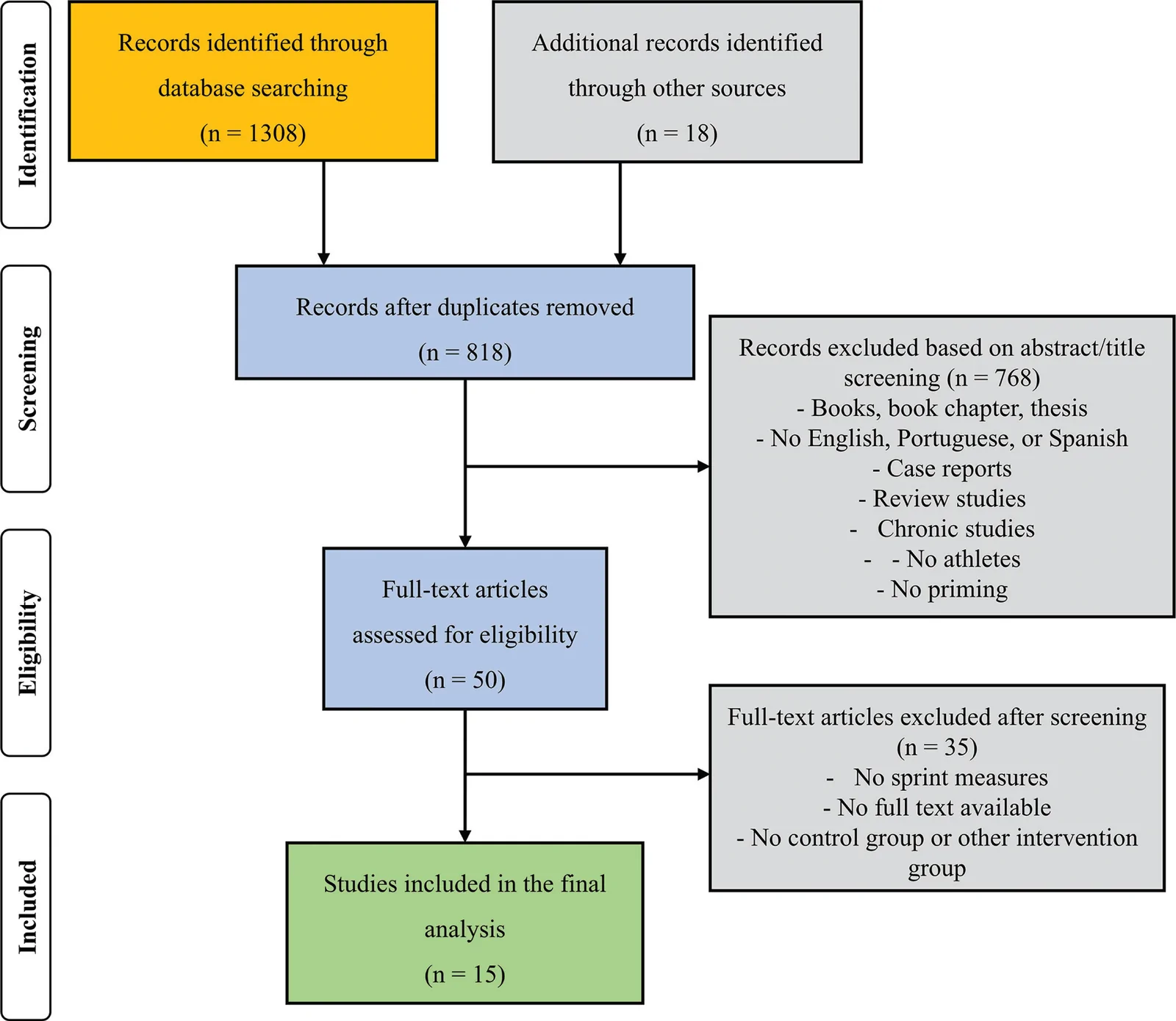
Flowchart of the study selection process.

**FIG. 2:**
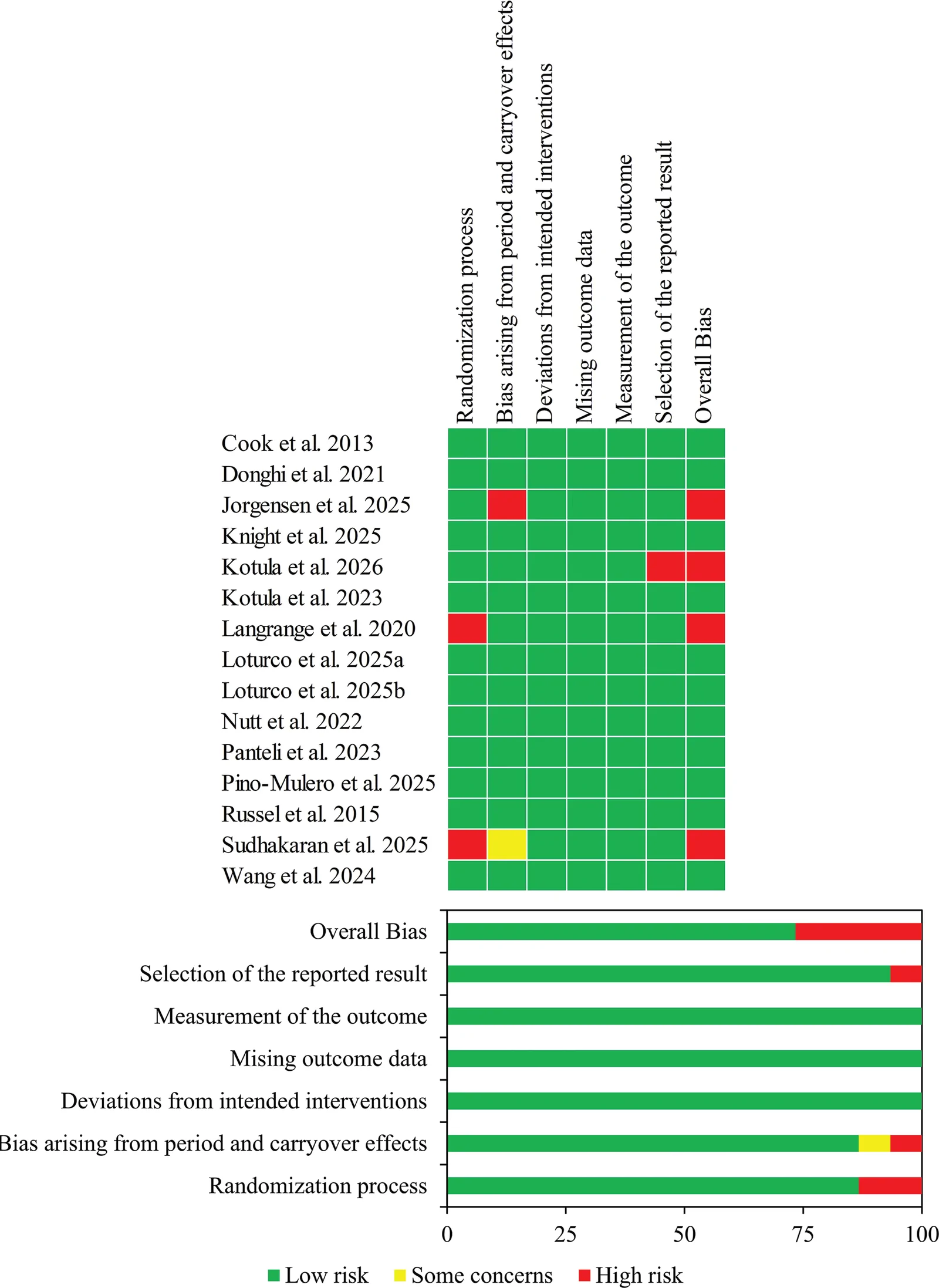
Risk of bias assessment of the included studies.

The methodological characteristics of the included studies are presented in Table 1. In total, 262 athletes from distinct sports disciplines were assessed, comprising 219 male and 43 female athletes. The priming protocols included assisted and resisted sprints, repeated sprint bouts, dynamic squats and deadlifts, loaded and unloaded jump squats, and plyometric exercises. The interval between the priming exercises and the sprint tests varied from 5.5 to 48 h. Sprint distances ranged from 20 to 50 m.

**TABLE 1:** Characteristics of the included studies.

Reference	n	Competitive level	Priming protocol	Rest interval	Sprint distance
Cook et al. 2014	18	Semi-professional male rugby union players	BP and BS: 3 × 50%, 3 × 80%, 3 × 90%, and 3 × 100% of 3RM or 5 × 40-m sprints	6 h	40 m

Donghi et al. 2021	12	Young male soccer players	4 × 12 HS, 6 speed ladder drills, 20-m sprints or 6 × 40-m repeated sprint running	5 h 30 min	40 m

Jorgensen et al. 2025	18	Elite young male soccer players	3 × 2 BS at 85% 1RM or 3 × 5 JS at 40% 1RM	20 h	30 m

Knight et al. 2025	25	Male rugby union players	3 × 3 BS at 85% 1RM and 5 × 3 JS at 40% 1RM	5 h 30 min	40 m

Kotula et al. 2026	12	Youth female sprinters	5 × 30-m sprints with 10% BM or 5 × 40-m sprints with 105–110% Vmax	24 h	50 m

Kotula et al. 2023	10	Female sprinters	4 × 40-m sprints with 10% BM or 4 × 40-m sprints with 105% Vmax or 2 × 40-m sprints with 10% BM and 2 × 40-m sprints with 105% Vmax	48 h	50 m

Langrange et al. 2020	41	Elite male midget or junior hockey players	5 × 5 HS at 85% 1RM + 6 SJ	6 h	40 m

Loturco et al. 2025a	21	Female rugby sevens players	6 × 6 JS at 40% or 80% 1RM	6 h and 24 h	40 m

Loturco et al. 2025b	20	Male rugby union players	6 × 6 JS at 40% 1RM or DJ at optimal RSI	6 h and 24 h	30 m

Nutt et al. 2022	16	Cricketers	1 × 4 trap bar deadlift at 50%, 70%, and 80% 1RM; 3 × 4 at 85% 1RM	5 h 30 min	35.6 m

Panteli et al. 2023	14	Well-trained male soccer players	4 × 8 JS; 4 × 8 jump shrugs; 3 × 6 DJ 30 cm or 2 × 4 15-m sprints; 2 × 4 DJ 30 cm + 10-m sprints	24 h	20 m

Pino-Mulero et al. 2025	16	Semi-professional young male soccer players	2 × 2 15-m sled push with 100% BM	24 h	20 m

Russel et al. 2015	15	Professional male rugby players	5 × 10 BP at 75% 1RM	5 h	40 m

Sudhakaran et al. 2025	8	Male collegiate badminton players	3 × 2 BP, BS, and deadlift at 85% 1RM	8 h and 24 h	20 m

Wang et al. 2024	16	Highly trained male national-level collegiate athletes	3 × 3 BS at 85% 1RM (with or without 30-s inter-repetition rest)	6 h	20 m

***Note*:** RM = repetition maximum; BM = body mass; BP = bench press; BS = back squat; HS = half squat; JS = jump squat; Vmax = maximal velocity; SJ = squat jump; DJ = drop jump; RSI = reactive strength index.

Figure 3 displays the forest plot illustrating the overall effect of pre- to post-intervention changes in sprint performance across the studies. Significant increases in sprint performance were observed after the priming protocols (SMD = 0.40 [95% CI: 0.22; 0.59]; Z = 4.23; *p* < 0.001; I^2^ = 5%). As shown in Figure 4, subgroup analysis revealed a significant improvement in sprint performance when the priming protocols were executed ≤ 8 hours before performance testing (SMD = 0.42 [95% CI: 0.21; 0.63]; Z = 3.90; *p* < 0.001; I^2^ = 0%). Meanwhile, for intervals ≥ 20 hours, no significant changes were observed (SMD = 0.27 [95% CI: -0.11; 0.65]; Z = 1.41; p = 0.16; I^2^ = 27%). In addition, no significant difference was observed between subgroups (Z = 0.84; *p* = 0.40). The 95% prediction interval ranged from 0.17 to 0.64 for the overall effect, from 0.23 to 0.60 for the ≤ 8 h subgroup, and from -0.29 to 0.79 for the ≥ 20 h subgroup.

**FIG. 3:**
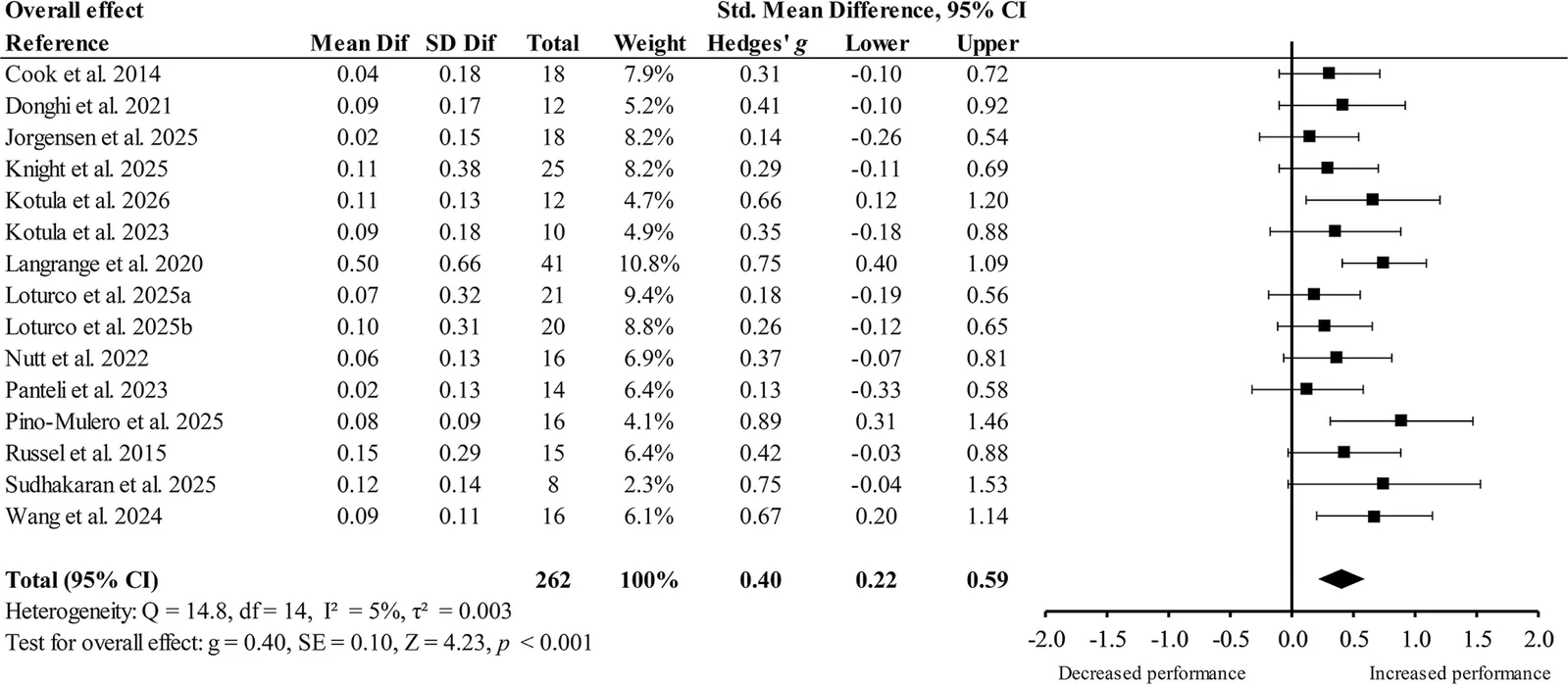
Forest plot showing the overall effect of priming protocols on sprint performance across the 15 included studies.

**FIG. 4:**
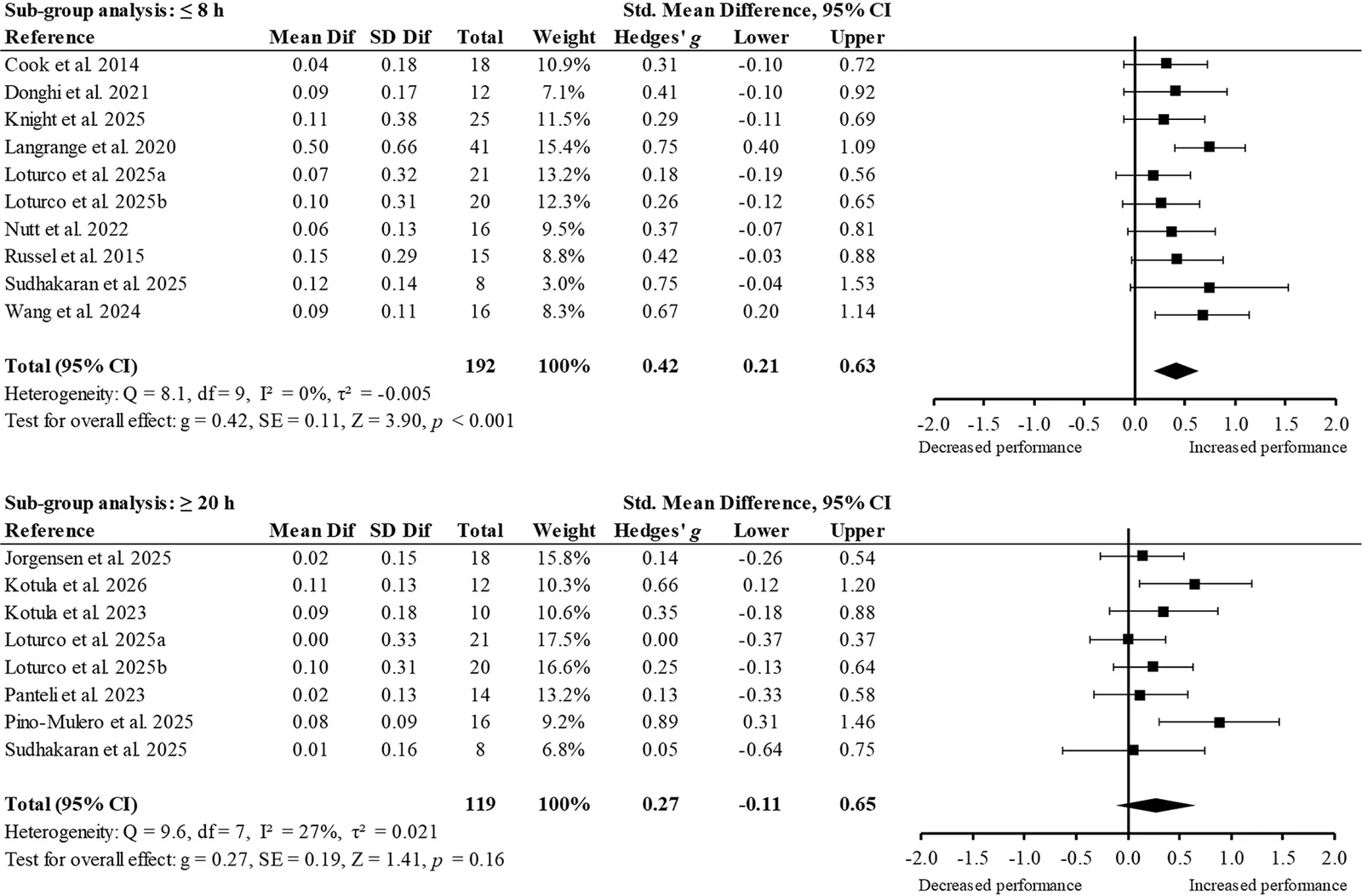
Forest plot of subgroup analyses across different time intervals showing the effects of priming protocols on sprint performance.

Sensitivity analyses varying the assumed correlation coefficient (*r* = 0.3–0.7) resulted in slightly higher heterogeneity estimates (an I^2^ range of 0–38%), but the magnitude and statistical significance of the pooled effect remained unchanged. Visual inspection of the funnel plot did not suggest substantial asymmetry. Egger’s regression test was not statistically significant (*p* = 0.24), indicating no evidence of small-study effects.

## DISCUSSION

This study aimed to assess the effectiveness of different priming strategies for enhancing the sprint speed of highly trained athletes. After a systematic review of the literature and a meta-analysis comprising 15 studies, we observed that, overall, priming exercises used as a pre-conditioning activity were effective in improving sprint performance in competitive athletes (SMD = 0.40), representing a small-to-moderate effect size with low heterogeneity (I^2^ = 5%). Subgroup analysis demonstrated that intervals between 5 and 8 h might be more effective for performance gains, but no significant differences were observed between the time intervals. The robustness of this result is confirmed by the 95% prediction interval, whose lower bound remained above zero for the overall effect, and by the sensitivity analysis, wherein the pooled effect did not change despite varying the assumed correlation. Importantly, the consistent overall effect, supported by the 95% prediction interval (i.e., 0.17 to 0.64), suggests that future studies conducted under comparable conditions are likely to observe beneficial outcomes of priming interventions, reinforcing the practical consistency and applied relevance of this relatively novel and increasingly adopted strategy. Although previous studies testing acute PAPE strategies or chronic variations in sprint performance have demonstrated minimal changes over time [3, 6, 11], the present findings indicate that sub-acute priming interventions (5–48 h) may represent a viable strategy to induce meaningful improvements in sprint performance in highly trained athletes.

The results confirmed the notion observed in previous narrative reviews that have shown the potential impact of priming activities for performance improvement in athletes from distinct sports disciplines [8, 9]. The main characteristics observed in the priming sessions are their low volume and the use of practical and applied training methods (e.g., light-to-moderate load ballistic exercises and sprint drills). This combination of high intensity (relative load or movement velocity) and low total volume appears to optimize neuromuscular stimulation while minimizing residual fatigue, which is particularly relevant in highly trained athletes with limited adaptive reserve [1, 5, 34, 35]. For example, Loturco et al. [14] demonstrated significant and similar effects of two priming protocols in which elite female rugby sevens players performed 6 sets of 6 jump squats at 40% or 80% of half-squat 1RM; 40-m linear sprint speed improved after 6 h in both conditions. Cook et al. [23] reported that a priming session consisting of 5 × 40 m sprints in semi-professional rugby union players improved 40-m sprint performance 6 h after the protocol. Collectively, these findings suggest that both resistance-based and sprint-based priming interventions may be effective when appropriately prescribed and individualized, thereby expanding their applicability across various training and competitive environments [8, 14, 23]. The results reported herein have important practical implications, as coaches can apply these strategies across diverse training and competition contexts, even in facilities with reduced equipment availability or under competition-day logistical constraints [8, 9].

The subgroup analysis revealed that intervals between 5 and 8 h might be more effective in inducing meaningful performance gains. The mechanisms associated with the effects of priming exercises are not yet fully understood [9, 32, 36]. Nonetheless, previous research has raised hypotheses regarding variations in hormonal responses and delayed neuromuscular activation [9, 32, 36]. For instance, Russell et al. [32] revealed that diurnal reductions in salivary testosterone levels were less pronounced after priming sessions compared with the control condition. Pratt [36] observed significant improvements in voluntary activation and “rate of torque development”, which are indirectly related to motor unit recruitment, discharge rates, and musculotendinous stiffness, 6 h after the implementation of an effective priming session, compared with the control condition. Although these findings may contribute to elucidating the mechanisms underpinning this delayed potentiation phenomenon, the limited number of mechanistic investigations precludes more robust and definitive conclusions [8, 32, 36].

The longer time intervals did not result in significant gains in sprint performance. The smaller number of subjects and studies analyzed for this subgroup might have contributed to this result. However, some individual studies have demonstrated improvements in sprinting speed and other physical performance markers [12, 31]. Kotula et al. [12] examined the effects of two distinct priming protocols on female sprinters, which involved 5 × 30-m resisted sprints with a load of 10% of body mass or 5 × 40-m assisted sprints at 105–110% of maximal sprint speed. As a consequence, 50-m sprinting speed increased significantly 24 h after both exercise interventions. Pino-Mulero et al. [31] observed significant improvements in vertical jump height and 20-m sprint performance 24 h after a priming session in which under-18 semi-professional soccer players performed 2 sets of 2 × 15-m resisted sprints with a load equivalent to 100% of body mass. Therefore, after analyzing 15 studies and dividing them into two subgroups, it appears that a relevant gap still exists regarding the use of priming exercises at longer time intervals (i.e., ≥ 24 h), regardless of the sport discipline or type of exercise implemented during the priming session.

Thus, future studies are needed to confirm these findings and further clarify the effectiveness of longer recovery intervals across different athletic populations and competitive contexts. This review is limited by the impossibility of comparing the effects of multiple priming strategies (e.g., resisted sprints vs. plyometrics, ballistics vs. nonballistic exercises; resisted vs. assisted sprints) on sprint performance. This limitation is due to the reduced number of studies testing similar exercise protocols. In addition, the effects of these methods still need to be understood in athletes from different competitive levels and age categories, as well as whether these strategies are more suitable for promoting performance enhancements in athletes from specific sport disciplines (e.g., team sports vs. track and field vs. racket sports). Nevertheless, this study revealed that, in general, priming exercises applied as a pre-conditioning activity were able to improve sprint performance across distances ranging from 20 to 50 m in highly trained athletes.

## CONCLUSIONS

The findings of this systematic review and meta-analysis indicate that priming strategies can be an effective method for enhancing sprint performance in highly trained athletes. Coaches and practitioners may implement low-volume, high-intensity priming activities such as ballistic exercises (e.g., jump squats with light-to-moderate loads), plyometrics, or short sprint drills to improve neuromuscular readiness prior to speed-oriented training sessions or competitive events. Based on the available evidence, priming sessions performed approximately 5–8 hours in advance appear to provide the most consistent benefits for sprint speed. These strategies may be particularly useful in competitive athletes, in whom further refinements in sprint performance are difficult to achieve through traditional training methods. It is essential to emphasize that the small-to-moderate effect size observed herein (SMD = 0.40) may correspond to an improvement of approximately 0.4–1.0% in sprint performance, based on the typical variability reported in highly trained athletes. This magnitude represents a meaningful change in elite contexts, considering that long-term performance gains are generally within ≈1–2%. Furthermore, priming protocols are typically characterized by low total volume, which helps stimulate neuromuscular function while minimizing residual fatigue. From a practical standpoint, these sessions can be easily integrated into pre-competition routines or speed-focused training days, even in environments with reduced equipment availability.

## References

[1] Haugen T, Tonnessen E, Hisdal J, Seiler S. The role and development of sprinting speed in soccer. Int J Sports Physiol Perform. 2014; 9(3):432–441. 10.1123/ijspp.2013-0121 23982902

[2] Loturco I, Bishop C, Zabaloy S, Moura TBMA, Ramos MS, Pereira LA, McGuigan MR. Variations in strength-speed-power performance across the season: do true changes occur in elite rugby players? Biol Sport. 2024; 41(3):201–211. 10.5114/biolsport.2024.135201 PMC11167459 38952901

[3] Loturco I, Fernandes V, Bishop C, Mercer VP, Siqueira F, Nakaya K, Pereira LA, Haugen T. Variations in Physical and Competitive Performance of Highly Trained Sprinters Across an Annual Training Season. J Strength Cond Res. 2023; 37(5):1104–1110. 10.1519/JSC.0000000000004380 36730012

[4] Haugen T, Seiler S, Sandbakk O, Tonnessen E. The Training and Development of Elite Sprint Performance: an Integration of Scientific and Best Practice Literature. Sports. Med Open. 2019; 5(1):44. 10.1186/s40798-019-0221-0 PMC6872694 31754845

[5] Haugen T. Sprint conditioning of elite soccer players: worth the effort or lets just buy faster players. Sport Perform Sci Rep. 2017; 1:1–2.

[6] Haugen TA, Solberg PA, Foster C, Moran-Navarro R, Breitschadel F, Hopkins WG. Peak Age and Performance Progression in World-Class Track-and-Field Athletes. Int J Sports Physiol Perform. 2018; 13(9):1122–1129. 10.1123/ijspp.2017-0682 29543080

[7] Boullosa D. Post-activation performance enhancement strategies in sport: a brief review for practitioners. Human Mov. 2021; 22(3):101–109.

[8] Pereira LA, Zmijewski P, Golas A, Kotula K, McGuigan MR, Loturco I. Priming Exercises and Their Potential Impact on Speed and Power Performance: A Narrative Review. J Hum Kinet. 2025; 98. 10.5114/jhk/204371 PMC12360937 40837515

[9] Harrison PW, James LP, McGuigan MR, Jenkins DG, Kelly VG. Resistance Priming to Enhance Neuromuscular Performance in Sport: Evidence, Potential Mechanisms and Directions for Future Research. Sports Med. 2019; 49(10):1499–1514. 10.1007/s40279-019-01136-3 31203499

[10] Valcarce-Merayo E, Latella C. Precompetition strategies to improve performance in endurance or repeated intermittent activity: Evidence and practical suggestions. Strength Cond J. 2023; 45(4):450–461.

[11] Loturco I, Pereira LA, Moura T, McGuigan MR, Boullosa D. Effects of Different Conditioning Activities on the Sprint Performance of Elite Sprinters: A Systematic Review With Meta-Analysis. Int J Sports Physiol Perform. 2024; 19(7):712–721. 10.1123/ijspp.2024-0005 38823792

[12] Kotuła K, Matusiński A, Maszczyk A, Pereira LA, Zajac A, Loturco I. Neuromechanical effects and predictive profiling of sprint priming strategies in elite youth sprinters. Biol Sport. 2026; 43:319–327. 10.5114/biolsport.2026.154150 PMC12884905 41668957

[13] Loturco I, Zmijewski P, Mercer VP, Moura TBMA, Ramos MS, Betelli MT, Pereira LA, McGuigan MR. Effects of Loaded and Unloaded Jumps Utilized as Priming Activities in Elite Male Rugby Players. J Strength Cond Res 2025.; 39(6):e781–e787. 10.1519/JSC.0000000000005093 40267473

[14] Loturco I, Zmijewski P, Mercer VP, Ramos MS, Betelli MT, Arenhart I, Moura TB, Pereira LA. Effects of light-load vs. heavy-load jump squats as priming activities in Olympic female rugby sevens players. Biol Sport. 2025; 42(2):85–94. 10.5114/biolsport.2025.142645 PMC11963119 40182726

[15] Wang Z, Yan B, Wang Y, Zhang C, Chen F, Girard O. A comparison of morning priming exercise using traditional-set and cluster-set configurations on afternoon explosive performance. Biol Sport. 2024; 41(3):129–135. 10.5114/biolsport.2024.133003 PMC11167474 38952900

[16] Harrison PW, James LP, McGuigan MR, Jenkins DG, Kelly VG. Prevalence and application of priming exercise in high performance sport. J Sci Med Sport. 2020; 23(3):297–303. 10.1016/j.jsams.2019.09.010 31594712

[17] Loturco I, Pereira LA, Mercer VP, Fernandes V, Moura T, Boullosa D. Warm-Up Methods Adopted by Brazilian Elite Sprint Coaches: Practical Insights From a National Survey. Int J Sports Physiol Perform. 2026, In Press. 10.1123/ijspp.2025-0394 41672056

[18] Moher D, Shamseer L, Clarke M, Ghersi D, Liberati A, Petticrew M, Shekelle P, Stewart LA. Preferred reporting items for systematic review and meta-analysis protocols (PRISMA-P) 2015 statement. Syst Rev. 2015; 4(1):1. 10.1186/2046-4053-4-1 PMC4320440 25554246

[19] Higgins JP, Altman DG, Gøtzsche PC, Jüni P, Moher D, Oxman AD, Savovic J, Schulz KF, Weeks L, Sterne JA. The Cochrane Collaboration’s tool for assessing risk of bias in randomised trials. BMJ. 2011; 343:d5928. 10.1136/bmj.d5928 PMC3196245 22008217

[20] DerSimonian R, Laird N. Meta-analysis in clinical trials. Control Clin Trials. 1986; 7(3):177–188. 10.1016/0197-2456(86)90046-2 3802833

[21] Cohen J. Some issues in power analysis: Effect size. In: Cohen J ed, Statistical power analysis for the behavioral sciences. Hillsdale (NJ): Lawrence Erlbaum Associates; 1988:531–535.

[22] Higgins JPT, Thomas J, Chandler J, Cumpston M, Li T, Page MJ, Welch VA. Cochrane handbook for systematic reviews of interventions. Chichester (UK): John Wiley & Sons; 2019. 10.1002/14651858.ED000142 PMC10284251 31643080

[23] Cook CJ, Kilduff LP, Crewther BT, Beaven M, West DJ. Morning based strength training improves afternoon physical performance in rugby union players. J Sci Med Sport. 2014; 17(3):317–321. 10.1016/j.jsams.2013.04.016 23707139

[24] Donghi F, Rampinini E, Bosio A, Fanchini M, Carlomagno D, Maffiuletti NA. Morning Priming Exercise Strategy to Enhance Afternoon Performance in Young Elite Soccer Players. Int J Sports Physiol Perform. 2021; 16(3):407–414. 10.1123/ijspp.2020-0094 33401241

[25] Jørgensen RB, Pedersen LT, Nielsen T, Egebæk HK, Jensen R. Delayed effects of heavy resistance or ballistic priming on sprint and agility in elite U19 football. J Sports Med Phys Fitness. 2025; 65(5):599–605. 10.23736/S0022-4707.25.16223-3 39982039

[26] Knight J, Russell M, Cunningham D, Cook C, Waldron M, Kilduff L. The effects of morning priming exercise on afternoon physical and cognitive performance in male rugby union players. bioRxiv 2025, doi: 2025.2005.2029.656928. 10.1371/journal.pone.0349645 PMC13268173 42296087

[27] Kotuła K, Matusiński A, Zając A, Krzysztofik M. Sprint Resisted and Assisted Priming for Peak Performance. J Strength Cond Res. 2023; 37(12):2354–2361. 10.1519/JSC.0000000000004557 37639672

[28] Lagrange S, Ferland PM, Leone M, Comtois AS. Contrast Training Generates Post-Activation Potentiation and Improves Repeated Sprint Ability in Elite Ice Hockey Players. Int J Exerc Sci. 2020; 13(6):183–196. 10.70252/ONUV8208 PMC7039519 32148640

[29] Nutt F, Hills SP, Russell M, Waldron M, Scott P, Norris J, Cook CJ, Mason B, Ball N, Kilduff LP. Morning resistance exercise and cricket-specific repeated sprinting each improve indices of afternoon physical and cognitive performance in professional male cricketers. J Sci Med Sport. 2022; 25(2):162–166. 10.1016/j.jsams.2021.08.017 34535402

[30] Panteli N, Hadjicharalambous M, Zaras N. Delayed potentiation effect on sprint, power and agility performance in well-trained soccer players. J Sci Sport Exerc. 2024; 6(2):131–139.

[31] Pino-Mulero V, Soriano MA, Giuliano F, González-García J. Effects of a priming session with heavy sled pushes on neuromuscular performance and perceived recovery in soccer players: a crossover design study during competitive microcycles. Biol Sport. 2025; 42(1):59–66. 10.5114/biolsport.2025.139082 PMC11694209 39758171

[32] Russell M, King A, Bracken RM, Cook CJ, Giroud T, Kilduff LP. A Comparison of Different Modes of Morning Priming Exercise on Afternoon Performance. Int J Sports Physiol Perform. 2016; 11(6):763–767. 10.1123/ijspp.2015-0508 26658460

[33] Sudhakaran SC, Dada T, Rajan R, Shafi M, Nisar NNS. Diurnal Post-Activation Performance Enhancement (PAPE) Effect of Resistance Priming in College Badminton Players. Polish Journal of Sport and Tourism. 2025; 32(1):8–13.

[34] Loturco I. Rethinking Sport Science to Improve Coach-Researcher Interactions. Int J Sports Physiol Perform. 2023; 18(11):1231–1232. 10.1123/ijspp.2023-0367 37793657

[35] Ronnestad BR, Mujika I. Optimizing strength training for running and cycling endurance performance: A review. Scand J Med Sci Sports 2014; 24(4):603–612. 10.1111/sms.12104 23914932

[36] Pratt JD. Investigation into the neuromuscular mechanisms of resistance priming. Queensland, Australia Queensland University of Technology; 2023:78 pages.

